# On the Management of Irregular Dentition

**Published:** 1848-07

**Authors:** J. B. Mitchell

**Affiliations:** Surgeon Dentist.


### ARTICLE V.

*On the Management of Irregular Dentition.* By J. B. MITCHELL, M. D., Surgeon Dentist.

IRREGULARITY of the second dentition, viewed in regard to its causes, is of two kinds: the one arising from a want of harmony between the development of the permanent set, and the decadence of the milk teeth, the other depending on a defect in the correspondence which ought to exist between the growth of the jaws and the increased volume of the second series of teeth.

Little difference of opinion at present prevails on the subject of the first kind of irregularity, the interference of the dentist being now usually limited to assisting the ordinary course of dentition, when it is tardy or over active, but in no way anticipating the operations of nature. The profession is much indebted to M. Delabarre and Mr. Thomas Bell, for the improved views now so generally entertained in regard to the management of this particular defect of dentition. The same unanimity, with the exception of some matters of detail, may also be said to exist in respect to the regulation of the teeth, in cases where either kind of irregularity has been allowed to become permanent.

That species of irregular dentition, however, which depends on disproportion between the capacity of the jaws and the size of the teeth, forms the subject of several conflicting opinions. Two principal views have been taken by dentists, the distinguishing features of which are, on the one side, a tendency to allow things to take their course until remedial measures are called for, and, on the other, a leaning to preventive means.

In the system which is based on the former view, reliance is almost exclusively placed on the natural expansion of the jaws during the second dentition—no decisive measure being adopted till after that period has elapsed, when, if room cannot be provided in the dental arch for the irregular teeth, by artificial means, they must be extracted. To convey a satisfactory idea of this system, I cannot do better than quote from the writings of some of its most eminent supporters. "When each tooth is only a little too large," says Delabarre, "we should, before employing the traction by threads, file between the several teeth: we may by this means obtain from the whole the space of a third or fourth of a tooth, which ordinarily is sufficient: but we should sooner sacrifice a tooth than cut the rest too much, when the size of these bones appears to be extraordinary, and, especially, if there exists the least defect in the conformation of the jaws. When the fault is very decided, I find it more prudent to remove the teeth out of the row than to attempt to bring them within the circle. However, we should never be in any haste to extract them, for the jaw sometimes increases when we least expect it, so that, instead of there being a sacrifice of several teeth which it was supposed would one day have to be removed, that of one only may be sufficient. While we are thus temporizing, therefore, we are only courting a favorable opportunity. It is consequently only after being perfectly satisfied that the teeth, which are badly arranged, can never be rectified, that I attempt to remove them; and I usually practise this operation about the age of twelve or thirteen, for then the anterior arch of the jaw is susceptible of but little increase. When the removal of some of the irregular teeth becomes necessary, there are some which we should extract in preference to others: thus, all dentists agree to sacrifice a lateral or central incisor which is wholly out of the row, and the same may be said of the canines, when in a similar position, unless the bicuspides be diseased." Mr. Bell to the same effect observes: "If the irregularity be very slight, and the want of space trifling, it will be sufficient to pass a very thin file between several of the teeth, so as not to deprive any one of them of the whole thickness of the enamel, and in this way a considerable space will be gained by the approximation of all the teeth so treated, and the irregular tooth be brought into its place by moderate pressure; but, if the want of space be so great as to afford no hope of its being remedied by this mode, it often becomes necessary to sacrifice one of the permanent teeth. It cannot be too strongly insisted on, that sufficient time and every possible encouragement should be afforded to allow of the expansion of the maxillary arch, before either of these operations is had recourse to; and I have often had reason to congratulate myself upon the result of having refrained from employing them, until the age of fourteen or fifteen years should have decided whether they would be ultimately necessary; at which period the arch of the jaw has been found to have expanded sufficiently to admit the irregular tooth into its place. . . If after the lapse of that period, during which the expansion of the jaw may have been expected to take place, an inferior incisor should be forced much out of its situation, it will generally be necessary to remove the irregular tooth itself; after which the others will approximate so as to close the vacancy, provided the teeth of the upper jaw do not interfere with this process. In the upper jaw, however, the removal of a central incisor would be too great a sacrifice; and it never, or scarcely ever, happens that such a step can possibly be necessary. The loss of a bicuspid, or, at the worst, of a lateral incisor—the latter may almost always be avoided—will be found sufficient."

It will be readily admitted that the principles involved in the above treatment are tantamount to a total abandonment of preventive measures; and that the long delay which is recommended is equivalent to trusting to chance for the obviating of a defective arrangement, instead of adopting means to prevent it. The character of this plan becomes the more apparent when we consider that, by the study of the constitution and predisposition of the patient, we have it in our power to predict, with considerable accuracy, what will be the ultimate development of the parts.

The fundamental errors of this system are its temporising nature and the sacrifices that are entailed by the delaying of the treatment. In the first species of irregularity, this delay is rather to be commended than condemned, but, in that kind of irregularity which arises from disproportion between the size of the teeth and the development of the jaws, I should consider the sacrificing of one of the permanent incisor or canine teeth, as little better than no treatment at all, or at least not such as one would expect from the superintendence of a professional man.

It was Mr. Fox who first developed the principles of the preventive treatment in these cases, and the practice, as laid down by him, affords a very clear statement of the means of managing this species of irregular dentition. The following are his very practical remarks on this subject: "Irregularity is often occasioned by the teeth being much too large for the space allotted to them, and then it will be necessary to remove one or more of the permanent teeth. When the incisores are perfectly regular, and the bicuspides have appeared before the cuspidati, there is so little space left that the cuspidati are thrust too much forward. It has been the common practice to permit the cuspidati to grow down a certain length and then to extract them. This operation certainly removes the deformity of projecting teeth, but it destroys the symmetry of the mouth, and takes away two teeth of great importance. The cuspidati are exceedingly strong; they form the support of the front of the mouth, and in the advanced periods of life, to those persons who have the misfortune to lose the incisores, they furnish an excellent means of fixing artificial teeth: on these accounts they should be preserved, and therefore it will be right to extract the first bicuspides on each side. The cuspidati will then fall back into the circle, and if there should be any vacant space, it will be so far back that no defect will be perceived. The first permanent molares often become carious soon after they appear; when this is the case, and the other teeth have not proper room, considerable advantage always attends their extraction. Their removal permits the bicuspides to fall back, and gives way for the regular position of the cuspidati. . . . If they be extracted before the second permanent molares appear, in a short time they will not be missed, because the bicuspides will go back, and the second and third molares will come forward, so that no space will be left. The front teeth may even derive much benefit from this gain of room as there will probably be left a small space between them, which will tend to their preservation; for it is observed, when teeth are situated so close as to press hard upon each other, they almost always fall into a state of decay."

Mr. Fox, then, is very decided as to the desirableness of providing space for the teeth in those cases where they are obviously disproportionate to the limited size of the jaws, by the removal of two or more of the back teeth, even should they be sound; and in this he is followed to a certain extent by Mr. Bell, who allows as we have seen above, that one, and even two bicuspides, may be removed for the purpose of making room, and further admits, that a decayed molar may sometimes be sacrificed to attain that object. He says: "As a general rule, it may be observed that in cases of the early decay of a first permanent molar, it will be proper to remove it if there should appear to be a want of room in the jaw, as the bicuspides will then be allowed to fall back and give sufficient space for the other teeth to come into their natural and regular situation. This, however, like all the other operations for remedying or preventing irregularity, should not be employed until the advance of the teeth in the front indicates an absolute tendency to an irregular position." The first sentence of this quotation is sound both in principle and practice, but it appears to me that what it does contain of good is almost neutralised by the closing caution, the only effect of which must be to render the measure in many cases nugatory. The case supposed is one in which there is crowding of the teeth from want of room in the jaw, and where the extraction of a decayed tooth would furnish the space required, if performed early enough. What must be the effects of delay in such a case till the teeth have actually made an abnormal advance in the front of the mouth? Clearly to preserve in the jaw an unsound, and, therefore, injurious tooth, which must ultimately be lost, but whose removal, if effected in good time, would provide the additional accommodation necessary, and to run the risk when it is extracted, after the teeth have advanced in the front of the mouth, that the space will be left permanently vacant instead of being profitably occupied. M. Delabarre also admits the propriety of providing space by the extraction of a small grinder if diseased, and hints at the removal of even a sound bicuspid in preference to sacrificing an irregular canine tooth; but he denies that the removal of a large grinder, under any circumstances, can be of use in favoring the arrangement of a canine tooth. His words are: "We should generally prefer to remove even one of these (bicuspides) in order to make room for a canine tooth which we believe can be brought back with facility. The evulsion of a first great molar affected with caries appears to me to be but little capable of favoring the arrangement of a canine tooth, though it may be useful in assisting that of a tardy bicuspid." It may be said, then, that all agree that the cases are not of unfrequent occurrence which demand the sacrifice of one or other tooth for the sake of preventing irregularity, or of removing it when established: it is only as to which is the proper tooth to select for this purpose, that any difference of opinion prevails.

When any of the permanent teeth are in a state of decay at the time the operation is demanded, the indication is obvious enough: the diseased teeth ought to be extracted and the sound left, taking care, however, to attend to the effect on the symmetry of the mouth. In such a case it will almost invariably be the first molar that requires to be removed; as about the age when irregularity commonly occurs, it is exceedingly rare to find any other of the permanent teeth affected with caries.

It is when the teeth are all sound that there is room for diversity of opinion; under these circumstances, the almost universal practice is to remove the first or second bicuspides. "It was the custom before Mr. Fox's work appeared," says Mr. Bell, "to remove the irregular tooth itself; but as the incisores and cuspidati are of far superior importance to the *bicuspides*, and as any partial vacancy which may remain is of much greater consequence near the front of the mouth than further back, it is much better, in all common cases, to sacrifice one of the *latter teeth*." "When from the arrangement I am led to believe that a small gap must result from the indispensable sacrifice of a tooth, I prefer to remove the second *bicuspides*, to facilitate the placing of a canine." (Delabarre's Second Dentition.) "When extraction is necessary [to make room in the jaws,] the *bicuspides* are the teeth that should be selected for this purpose, and usually the second should be preferred to the first." (Dr. Arms, in *American Journal of Dental Surgery*, vol. v, p. 215.) "Whenever the space in the jaws appears too much confined, * * * one or more of the *bicuspides* may with incalculable advantage become a sacrifice." (Parmly's Lectures, p. 85.) "The teeth which are decayed should be extracted; but if all the dental organs are sound, we should remove the first *bicuspides*." (Garriol on the Diseases of the Mouth, p. 112.)

Mr. Koecker is the only writer that I am acquainted with who advocates the extraction of the first large grinders in preference to the bicuspides or other teeth: this author has gone minutely into the subject, and his views are particularly worthy of attention. "Those teeth," says Mr. Koecker, in his "Principles of Dental Surgery," "which are most subject to decay, least important, and the removal of which would afford the most relief to the whole set, are the proper ones to be chosen for extraction. As the loss of the incisors and cuspidati greatly disfigures the set, they ought to be preserved if possible; and I have hardly ever seen a case in which it was necessary to extract any of them with the view to give room to the rest, where an early attention had been paid to the state of the teeth. The preservation of the bicuspides, also, should be a matter of particular consideration; and the usual practice of extracting the first bicuspid teeth, to make room for the cuspidati, ought to be avoided, as well as the removal of the lateral incisores, by an early treatment.

"The first molares are generally most predisposed to disease; they are least important as regards both appearance and utility, and so situated as to afford, by timely removal, sufficient room for the anterior teeth, as well as for the second and third molares. If these teeth are extracted at any period before the age of 12 years, all the anterior teeth will grow more or less back, and the second and third grinders so much towards the anterior part of the mouth as to fill up almost entirely the vacant spaces caused by the removal of the first molares. In almost every instance all irregularity will be prevented by this treatment, and all the teeth will take a proper position. But besides this advantage, another more important benefit will invariably follow, viz. all the teeth will be improved in strength and health, and particularly the *dentes sapientiæ*, which will sometimes penetrate the gums much sooner, and prove of larger size, and possessed of greater firmness than usual."

In order more fully to elucidate this subject, let us give an attentive consideration to the distinctive characteristics of the first permanent grinders. These teeth are the only ones of the second set in which ossification has commenced at the period of birth; they are, therefore, the first formed of all the adult teeth. At the age of twelve months the ossification of the first permanent molares is pretty well advanced, while the second molares are usually found as mere shells at the completion of the first dentition. Such a vast difference in the period of formation of the first and second molares is a remarkable circumstance; but if we turn our attention to the time of cutting of these teeth, we shall be still more struck with the individual peculiarities that are severally exhibited by them. While the second persistent grinders are not generally cut before the twelfth year, the first four grinders make their appearance about the age of six, before the milk-teeth begin to be shed; so that about twelve it is not unusual to meet with children who are furnished with twenty-four teeth, twenty of which, of course, are deciduous. This very early appearance of the first molares exercises considerable influences on their characters and constitution. Interposed, as it were, between the first and second set of teeth, they would appear almost to occupy an intermediate place between the two series. On this account some have recognised in them an analogy to the wisdom-teeth, and have termed them the wise milk-teeth. In conformation and mode of growth the three persistent grinders agree; but in regard to the time of their appearance the greatest diversity exists. With no fewer than seven years between the ages when they appear, each of these three teeth may be said to belong to a distinct septenary period.

It is matter of every-day observation that the first molares, both in the upper and lower jaw, are the teeth which are most subject to decay: indeed, there are very few adults in whom they are perfectly sound; and in a large proportion of children above eleven, decay will be found to have commenced in them. The statistics of extraction furnish corresponding results. Mr. Tomes, in his Lectures, published in the Medical Gazette, states, that out of 1736 teeth extracted at the Middlesex Hospital, 642 were first molares, being no less than 36 per cent. It is also often remarked that these teeth, from their first appearance, are of a dull or slightly bluish color, very different from the complexion of healthy teeth, and uniformly indicative of incipient decay—an observation which Dr. Ashburner has had occasion to record in many of the cases of anormal development and crowding of the permanent teeth given in his very instructive work on Dentition, and which might also be extended to the wisdom-teeth. This defective constitution and predisposition to decay in the first molares may be readily accounted for by a reference to the comparatively early period of their formation, and their consequent exposure to the effects of those derangements of health which are so frequent in infancy, and especially during the first dentition, as well as from their being in the mouth during the whole time of the shedding of the temporary set, when they are almost certain to be neglected, if not injured, by being made to perform offices for which they are not calculated. M. Serres has noticed the influences which so specially bear on those teeth: "J'observerai, comme je l'ai deja dit, que toas les grands efforts portent sur les grosses molaires, et notamment sur la première.

These considerations ought to be sufficient to determine the extraction of the first molares in preference to all the other teeth, for the purpose of fulfilling the indication presented by the species of irregularity under discussion. But there is an additional reason for adopting this practice in the peculiar position the first molar teeth occupy. Situated in the centre of the half circumference of the jaw—which it ought never to be forgotten is composed of two symmetrical members—they hold a place between those permanent teeth that succeed the temporary set and those that are superadded, both of which series form foci of irregularity, tending, the one forwards from the first molar as a fixed point towards the front of the mouth, the others backwards, towards the ascending ramus of the jaw. That irregularity to which the latter—namely, the superadded grinders, are exposed, exhibits itself always at the period of the cutting of the wisdom-teeth, in the same manner that the irregularity of the former series, which the French happily enough denominate *dents de remplacement*, generally shows itself at the time the canine teeth appear. Anormal development of the wisdom-teeth, which depends on the want of space between the coronoid apophysis and the second large grinder, has attracted more attention from medical practitioners than that of the other teeth, on account of the sympathetic effects it gives rise to being very often of a violent and extraordinary character. Among Dr. Ashburner's cases are some very remarkable ones of this irregularity, and every practical dentist is well acquainted with its nature and effects. The usual practice in such cases is to remove the irregular teeth themselves when they can be laid hold of, and, failing in that, the second permanent grinders. But this will be found unnecessary if, in cases of want of consentaneous development between the teeth and jaws, the practice of Koecker be followed; for the extraction of the first molares gives relief both forwards and backwards, and when it is early had recourse to, the occurrence of irregularity will be entirely prevented, and the health of the wisdom-teeth guaranteed.

The truth of these remarks cannot be better illustrated than by the following case from Dr. Ashburner's treatise: A shoemaker, aged 22, suffered from tic douloureux, stammering, and confusion of ideas, accompanied with obstinate constipation. "The jaws were small; the four last molares were wanting; the spaces for them were small. In the upper jaw the tubercles were prominent behind the second molares; in the lower jaw there was not room for the *dentes sapientiæ.* The first molar teeth had each specks in them, and were of a more blue tint than the others. With a conviction on my mind that this man was suffering from a retarded and obstructed development, I knew not how to afford him relief. If that part of each jaw which contained the germs of the wise teeth could grow faster, there would be room for the teeth to come through: but how was the tendency to growth to be given to them? Going into the country, where a pure air would invigorate him, and make him expand his frame, was out of the question. To give him iron and bark and other corroborants, in town, would do little good; but combined with an eccoprotic and alterative course, it was the only plan left: for to remove the four healthy teeth which prevented the egress of the wise teeth was an unwarrantable experiment. This man came backwards and forwards to me for several months. I varied his medicines: I gave him colchium with rhubarb, and directed him to sponge his body with warm vinegar. Nothing relieved him; and I lost sight of him for fifteen months. He had been in the neighborhood of Birmingham with some relations; he had gained flesh, and looked more healthy and had lost the tic and the twitchings. The jaw had increased, and he had cut the upper wisdom-teeth; but the lower ones were not through, there was still too small a space. He had suffered much from decayed teeth, and had had three drawn, and there were four decayed teeth and a stump still remaining. The pressure from behind in the lower jaw had wrought a remarkable change in the incisor teeth. When I first saw them they were even; now they were huddled together, and lapped over one another. Nature had in this case made greater havoc with the teeth than I should have done had I not regarded the removal of four healthy teeth an experiment I had no right to make." (P. 143.) In this most instructive case there can be no doubt but that the timely removal of the four specked first molares would have effectually relieved the pressure both before and behind, and thus prevented the havoc which Dr. Ashburner, with so much ingenuousness, bewails; and that without the loss of a single sound tooth.

One other case from the same source I cannot refrain from citing. It is that of a girl aged 15, who had spasms of the muscles of the neck, and other nervous symptoms amounting almost to paralysis. (Dentition and some coincident Disorders, pp. 208-11.) "This young lady's teeth being very unusually large, and the jaws not growing with sufficient rapidity for the space required by the coming teeth, so much pressure operated to keep them back that they could not come through. Under the idea that the developing teeth, which appeared to be advancing most rapidly in the lower jaw, required space, the two second molares were extracted. The benefit was not striking, but a burning pain, and sense of weight on the top of the head, about the frontal and parietal bones, becoming very urgent, it was thought advisable, on the 16th of September, to remove the corresponding second molar teeth in the upper jaw. The operation was succeeded by immediate relief to the head, and a partial restoration of the power of moving the fingers of the left hand." The patient, however, did not at once get rid of all her painful symptoms, and Dr. Ashburner, with an impartiality which is as laudable as it is rare, closes the case with the following acknowledgment: "Had I been bold enough to sacrifice the four molar teeth which obstructed the development of the wise teeth at an earlier period, I feel convinced that I should have saved this young lady a world of pain and other evil." Undoubtedly such would have been the result, but it would have been preferable practice to extract the four first instead of the four second grinders.

I often meet with patients who, after having had one or more of the bicuspides removed to afford room in the jaw, have, a few years later, lost the first molares from caries; thus incurring a double loss, which in all probability would have been prevented by sacrificing the large instead of the small grinders. It is not uncommon, also, to see adults in whom the wisdom-teeth are so much out of their natural position as to be entirely useless, while the vacant spaces left by the first molars, lost from decay, attest that if they had been extracted early, the wise teeth would have had room to grow forward; so that eight grinders would have been secured for use instead of only four or five, and no perceptible void left.

In deciding the question as to the sacrificing of the bicuspides or the first molares, it is not unimportant to recollect that about the time when it generally is desirable to perform the operation, the former have just appeared and are quite new teeth, whereas the latter have already been in use five or six years, and are, by that length of time, older teeth.

